# Author Correction: Miniaturization during a Silurian environmental crisis generated the modern brittle star body plan

**DOI:** 10.1038/s42003-022-03067-8

**Published:** 2022-02-02

**Authors:** Ben Thuy, Mats E. Eriksson, Manfred Kutscher, Johan Lindgren, Lea D. Numberger-Thuy, David F. Wright

**Affiliations:** 1 Natural History Museum Luxembourg, Department of palaeontology, 25, rue Münster, 2160 Luxembourg, Luxembourg; 2 Department of Geology, Lund University, Sölvegatan 12, SE-223 62 Lund, Sweden; 3 Dorfstrasse 10, 18546 Sassnitz, Germany; 4 Department of Paleobiology, National Museum of Natural History, Smithsonian Institution, Washington, DC 20013–7012 USA; 5 American Museum of Natural History, Division of Paleontology, Central Park West at 79th St, New York, NY 10024 USA

**Keywords:** Palaeontology, Evolution, Taxonomy

Correction to: *Communications Biology* 10.1038/s42003-021-02971-9, published online 10 January 2022.

The original version of this Article contained errors in Fig. 1 and Supplementary Fig. 2 in which the ages of the stratigraphic chart were stated in ascending order as 419, 423, and 428 Ma. The corrected values are 423, 427, and 433 Ma, respectively, and have been replaced in Fig. 1 and Supplementary Fig. 2. This error also appeared in the third paragraph of the Introduction, which incorrectly read “…that range in age from the Telychian (latest Llandovery, about 428 Mya) to Ludfordian (late Ludlow, about 420 Mya).” The text should read “…that range in age from the Telychian (latest Llandovery, about 433 Mya) to Ludfordian (late Ludlow, about 423 Mya).” The errors have been corrected in both the PDF and HTML versions of the Article.

## Supplementary information

Updated Supplementary Information

