# Supplementary material for: Miniaturization during a Silurian environmental crisis generated the modern brittle star body plan
Source: Commun Biol. 2022 Jan 10;5:14. doi: 10.1038/s42003-021-02971-9 (PMC8748437; doi:10.1038/s42003-021-02971-9)
Supplement: Supplementary file 2 — Supplemenary Information [file 42003_2021_2971_MOESM2_ESM.pdf]

Supplementary information for "Miniaturization during a Silurian environmental crisis generated the modern brittle star body plan"

Thuy, B., Eriksson, M.E., Kutscher, M., Lindgren, J., Numberger-Thuy, L.D. & Wright, D.F.

Supplementary Methods: List of characters used to score the data matrix for the Bayesian-inference analysis:

1. Dorsal disc covering: (0) no scales, (1) thin scales, (2) thick scales.
2. Disc integument extending to arm tips: (0) no, (1) yes.
3. Central primary plates: (0) indistinguishable, (1) identifiable.
4. Primary radial plates: (0) indistinguishable, (1) identifiable.
5. Disc granules or spines: (0) none, (1) present.
6. Disc granules/spines extension: (0) sparse, (1) forming dense cover.
7. Radial shields: (0) absent, (1) present.
8. Radial shields length: (0) less than one third of the disc radius, (1) between one third and half of the disc radius, (2) more than half of the disc radius.
9. Radial shield shape: (0) scalene triangular, (1) isoscele triangular, (2) half-circle.
10. Exposure of radial shields: (0) central part to almost entire radial shield exposed, (1) distal portion of radial shield exposed, (2) distal-adradial portion of radial shield exposed.
11. Genital plates: (0) absent, (1) present.
12. Shape of abradial genital plate: (0) paddle-shaped, (1) sabre-like with longitudinal rim, (2) sabre-like with widened distal portion, (3) sabre-like without ridge or rim, (4) half-ring shaped, (5) scale-like, wide, with central longitudinal ridge.
13. Shape of adradio-distal tip of abradial genital plate: (0) straight or convex, (1) concave.
14. Genital plates bearing papillae: (0) no, (1) yes.
15. Marginal disc plates: (0) absent/indistinguishable, (1) recognizable.
16. Oral shields: (0) absent, (1) present.
17. Madreporite: (0) unrecognizable, (1) clearly identifiable.
18. Oral papillae s.l.: (0) absent/unrecognizable, (1) present.
19. Teeth: (0) absent/unrecognizable, (1) present.
20. Oral plates: (0) separated in two parts, (1) fused into a single plate.
21. Abradial muscle fossa of oral plate: (0) central depression, (1) large, well defined flange.
22. Adradial muscle attachment area of oral plate: (0) ventral, lining ventral or ventro-distal edge of articulation area, (1) in middle position, vertical, (2) with large, dorsal, spoon-shaped depression.
23. Integument of arms: (0) naked, (1) bearing granules, spines or disc scales at least in proximal segments.
24. Ventral arm plates: (0) absent, (1) present.
25. Proximal ventral arm plates in contact: (0) no, (1) yes.
26. Dorsal arm plates: (0) absent, (1) present.
27. Number of dorsal arm plates: (0) single, (1) several.
28. Proximal dorsal arm plates in contact: (0) no, (1) yes.
29. Lateral arm spines: (0) absent, (1) present.
30. Lateral arm spines orientation: (0) predominantly parallel to arm axis, (1) erect.

31. Length of longest lateral arm spine : (0) shorter than half an arm segment, (1) between half an arm segment and one segment, (2) between one and two arm segments, (3) longer than two arm segments.
32. Lateral arm spines in cross section: (0) round, (1) flattened.
33. Lateral arm spine tip: (0) pointed, (1) blunt.
34. Ambulacral arm spines: (0) absent, (1) present.
35. Ambulacral arm spine shape: (0) spine-like, (1) scale-like.
36. Tentacle scales: (0) absent, (1) present.
37. Ambulacral plates: (0) separate, (1) fused.
38. Ambulacral plate pattern: (0) alternating, (1) opposite.
39. Lateral articulation of ambulacral plates: (0) peg, (1) vertical ridge, (2) peg and ridge.
40. Zygocondyle orientation: (0) almost horizontal, arched, (1) oblique, straight, (2) almost vertical, straight.
41. Zygosphene: (0) absent, (1) present.
42. Zygosphene position: (0) at same level as zygocondyles, (1) ventral with respect to zygocondyles.
43. Auluroid canal: (0) completely enclosed by ambulacral plates, (1) partly open, (2) completely open.
44. Sublateral arm plates: (0) separate, (1) not recognizable as separate plates.
45. Lateral arm plate position: (0) ventral to subventral, (1) lateral.
46. Lateral arm plates meeting dorsally in proximal to median arm segments: (0) no, (1) in most arm segments.
47. Lateral arm plates meeting ventrally in proximal to median arm segments: (0) no, (1) in most arm segments.
48. Lateral arm plates with constriction: (0) no, (1) yes.
49. Ventral portion of lateral arm plates projecting ventro-distalwards: (0) no, (1) yes.
50. Ventro-distal tip of lateral arm plate projecting ventralwards: (0) no, (1) yes.
51. Outer surface stereom of lateral arm plates: (0) without ornamentation, (1) with enlarged trabecular intersections, (2) with vertical striation, (3) coarsely meshed, reticulous.
52. Outer proximal edge of lateral arm plate with band of differentiated stereom: (0) no, (1) yes.
53. Outer proximal edge of lateral arm plate with spurs: (0) no, (1) yes.
54. Lateral arm spine articulations: (0) absent, (1) present.
55. Lateral spine articulations position: (0) on same level as outer surface stereom, (1) on elevated ridge, (2) in notches of the distal edge.
56. Lateral spine articulations separated from distal edge of lateral arm plate: (0) no, directly on distal edge, (1) yes, by regular stereom, (2) yes, by a differentiated band of stereom.
57. Muscle and nerve openings separated: (0) no, single opening, (1) yes.
58. Muscle and nerve openings encompassed by dorsal and ventral lobes: (0) no, (1) yes, (2) muscle opening encompassed by an elevated ring.
59. Dorsal and ventral lobes straight: (0) no, at least one bent, (1) yes.
60. Dorsal and ventral lobes parallel: (0) no, shifted, (1) yes.
61. Dorsal and ventral lobes separated: (0) yes but without knobs, (1) yes, by one or several knobs or denticulate stereom, (2) no, merged at their proximal tips.
62. Orientation of dorsal and ventral lobes: (0) nearly horizontal, (1) tilted, (2) nearly vertical.
63. Single opening encompassed by: (0) elevated ring, (1) two comma-shaped lobes, (2) simple ridges, (3) dorsalwards pointing chevron.

64. Muscle and nerve openings separated by: (0) small ridge, (1) large prominent ridge or regular stereom.
65. Ambulacral spine articulations: (0) absent, (1) present.
66. Ventral edge of lateral arm plate lined by ridge: (0) no, (1) yes.
67. Lateral arm plate with tentacle notch: (0) no, (1) yes.
68. Articulation between ambulacral plate and lateral arm plate: (0) via sublateral arm plate, (1) with simple peg, (2) with ridge, (3) with knobs, (4) with peg and ridge.

Supplementary Methods: Data matrix used for the Bayesian-inference analysis:

Aganaster gregarius 10110N10101??00101110001010010001 0N1111211211111111101011 2NNNNN10112
Eugasterella thorni 1000110NNN0NNN0000000010N0NN1020011000001001100000 1??1010NNNNN1N1001
Strataster ohioensis 1000110NNN0NNN0010100010N0NN112001100000??011000001??1000NNNNN1N1001
Pradesura jacobi 1000100NNN0NNN001000??00N0NN111001000000??001000000??1110NNNNN2N1000
Ophiaulax decheni 00110N0NNN1200110001??010100100000N111111111111110111??10112NNNNN10112
Stephanoura belgica 10000N1010120011000 1??010100112000N11111112111111111?11112NNNNN10112
Ophiurina armoricana 00000N0NNN0NNN100001??010110110??0N011????211010111??1110NNNNN0N011?
Ophiurina lymani 0000100NNN0NNN100001??00N100110000N011????211111113??1110NNNNN0N011?
Lapworthura miltoni 1000100NNN0NNN001010??00N0NN11300100010???011001000??1110NNNNN0N1001
Hallaster cylindricus 10000N0NNN0NNN000010??00N0NN112001000100100110010001 01110NNNNN0N1001
Furcaster leptosoma 10000N0NNN0NNN0000100000N0NN113001101121100110000031 01220NNNNN3N1004
Eospondylus primigenius 10000N0NNN0NNN001000??00N0NN11300100112111010001003101200NNNNN3N1004
Muldaster haakei ??????????????????????00N0NN111000N0110111111011111101020NNNNN0N0111
Ophiopetagno paicei ????????????????????????????11??????110111011??1111101020NNNNN0N1111
Onychaster flexilis 10000N0NNN0NNN000010??00N0NN101111001120100100000030 01200NNNNN3N100?
Vandelooster plicatilis 01000N0NNN0NNN000010??00N0NN0NNNN110000???011000001000NNNNNNNNNN1001
Cheiropteraster giganteus 0100100NNN0NNN000010??00N0NN0NNNN10000000N011000001000NNNNNNNNNN1001
Crepidosoma wenlocki 10000N0NNN0NNN1000?0??10N0NN0NNNN0N0000???011000001000NNNNNNNNNN0001
Encrinaster goldfussi 1000100NNN0NNN100010??00N0NN0NNNN0N000000N011000001000NNNNNNNNNN0001
Ophiura ophiura 10110N111015010101110001010110111 0N111121121101000111 12010NNNNN10012
Ophiocten sericeum 10110N101015010101110001010111 2000N11112112110100021112010NNNNN10012
Ophiothrix fragilis 10111012021410010011120111011130 00N11112112110001011011011 0121N00013
Ophiopholis aculeata 10101011021410011111120111111120 00N111121121100000110110110121N00013
Ophiacantha bidentata 10001111111130001011100010100113000N111121121111100211111110020N00012
Ophiocomina nigra 10001110111310011111000111011130 00N111121121101110210111110020N00012

Supplementary Methods and Discussion: Tip-dating phylogenetics, sensitivity analyses, and methods for empirically calibrating prior distribuitons

All MrBayes scripts and R code to reproduce the analyses are available at: https://doi.org/10.5281/zenodo.5619312

Description and explanation of prior distributions in tip-dated phylogenetic analysis

The following prior distributions were specified in the Bayesian tip-dated analysis presented in the main text. The complete NEXUS file with all commands, fossil ages, and topological constraints is available at the github link above.

Morphological model:

Lset nbetacat=5 rates = lnorm Coding=informative;
Prset symdirihyperpr = fixed(infinity)

A basic Mk model (Lewis, 2001) with lognormally distributed variation among morphologic 'sites'/characters. We chose a lognormal distribution to model among site variation based on results from a modeling study by Wagner (2012) that found morphologic characters from fossil datasets often have a slightly better fit to lognormal distributions over gamma distributions.

Tree age:

prset treeagepr= Uniform(485.4, 521);

This prior spans a fairly wide interval and is constrained by evidence from the known echinoderm fossil record. Broadly speaking, it assumes that ophiuroids diverged from other major lineages of echinoderms sometime between the middle Cambrian (Miolingian) to the start of the Ordovician Period.

Clock model:

Prset clockratepr = Normal(0.0025, 0.1);
Prset clockvarpr = igr;
Prset igrvarpr = Uniform(0.0001, 200);

These commands implement an independent gamma rates (IGR) relaxed clock model. The priors for base rate of the clock and the variance of the IGR process were chosen to be broad, vague, and span multiple orders of magnitude following the recommendations in Matzke and Wright (2016). Alternatives to these priors were explored and are discussed in the section on sensitivity analyses below.

Diversification and fossil sampling priors of the FBD model:

Prset speciationpr = Uniform(0.01, 10);
Prset extinctionpr = Beta(1,1);
Prset fossilizationpr = Beta(2,2);

The "speciation" prior, which actually represents net diversification in MrBayes, was chosen because it's a broad, vague prior that spans several orders of magnitude (Matzke and Wright, 2016). The extinction prior was chosen to be a Beta(1,1) because it's a "flat" distribution, which corresponds to a uniform distribution between values of 0 and 1. We chose a Beta(2,2) prior to model fossilization because it has a symmetrical, convex shape with a maximum at 0.5 and decreases toward boundary values of 0 and 1. Based on our intuition of echinoderm fossil sampling rates, and previously published studies (e.g., Foote and Raup, 1996), it seemed more reasonable *a priori* that fossil sampling rates would be more likely to be a value closer to the center of the distribution than values closer to the boundaries. An alternative, empirically informed approach to specifying the fossil sampling prior was also explored. The details of that analysis are discussed below.

Sensitivity analyses for tip-dated phylogenetic and macroevolutionary inferences

To assess whether our phylogenetic and macroevolutionary results are sensitive to the assumptions and prior distributions, we conducted six additional tip-dating analyses that vary in choice of one or more of the following: FBD priors, constant vs. time heterogeneous rates of sampling and diversification, whether or not the sampling rate prior was empirically informed, and whether or not clock model priors were calibrated to the dataset. Supplementary Table 1 depicts how these sensitivity analyses differ from those presented in the main text (i.e., "main analysis") and from one another. Sensitivity analyses are labeled as A-1 to A-6. A brief sketch of each analysis is provided below.

<u>Note</u>:
Sensitivity analyses labeled A-3 to A-5 incorporate information from empirical estimates of fossil sampling obtained from analysis of fossil ophiuroid data obtained from the Paleobiology Database (PBDB) (paleobiodb.org). Thus, these sampling estimates are independent of the phylogenetic methods used in the main analysis, and allow for FBD analyses to be informed by the much greater number of Phanerozoic fossil occurrences than could be operationally included in our tip-dating analysis. This approach has the advantage of combining inferences from large-scale taxonomic occurrence databases, which have an advantage in terms of sample size, with more taxonomically focused studies using phylogenetic methods. Moreover, the incorporation of empirical constraints on parameter values and/or prior distributions may be critical for reducing parameter identifiability issues of birth-death-sampling models (Louca et al., 2021). A more detailed explanation of the methods used to estimate these values from PBDB data and how they were used specify fossil sampling priors is provided in the next section.

A-1. All priors were identical with those of the "main analysis", but with the tree topology left unconstrained rather than fixed to be the topology obtained from the undated analysis (i.e., testing whether tip-dated analysis recovers a similar phylogeny to the undated analysis).

A-2. This analysis assumed a constant rate FBD process applied to the whole Phanerozoic instead of the time-heterogeneous model estimating different piecewise-constant rates for each geologic stage. This analysis dramatically reduces the number of parameters estimated (but at the expense of biological plausibility).

A-3. All priors were identical to those of the main analysis except for fossil sampling. In this analysis, we fixed the fossil sampling rate to be equal to an empirically-based estimate

corresponding to the mean sampling rate of fossil ophiuroids estimated using PBDB data (see next section for how this was calculated).

A-4. Similar to A-3, all priors were identical to those of the main analysis except for fossil sampling. In this analysis, we used the mean and variance of an empirical distribution of fossil sampling rates for fossil ophiuroids spanning the Phanerozoic to specify Beta distribution as a prior on the FBD sampling rate. The hyperparamters of this Beta distribution were chosen in a way to ensure the distribution has the same mean and variance of the empirical distribution (see discussion below).

A-5. Identical to A-4, but with the tree topology left unconstrained instead of fixed.

A-6. This analysis tested whether our results were sensitive to variance in clock model priors. Instead of using a broad, uniform prior for the variance parameter of the IGR model, we calibrated this prior to our data using the method described by Ronquist et al. (2012). Using the scripts provided in the supplemental data file of Ronquist et al. (2012), we set the prior on the IGR variance parameter to be an exponential distribution parameterized by the slope obtained from a linear regression on branch lengths estimated under a non-clock model and a strict clock analysis. We also calibrated the prior on the base rate of the clock. Again, following methods described in Ronquist et al. (2012), we divided the median posterior tree height from an undated analysis by the oldest plausible age of the oldest fossil in the analysis (i.e., 485.4 Ma) to obtain an estimated clock rate, which was used as the mean of a lognormal prior of the clock rate, and the upper 95% credibility estimate of tree height was used to obtain a standard deviation for the lognormal distribution (Ronquist et al., 2012).

Results:

We compared the results of all six sensitivity analyses to our initial "main analysis" by visually inspecting topologies of their resulting 50% majority-rule trees and calculating the frequencies (i.e., posterior probability) that *Ophiopetagno paicei* and *Muldaster haakei* were placed as sampled ancestors in the posterior distribution of trees. Despite the vast differences in assumptions and prior distributions used in these analysis, all six recovered identical phylogenetic positions, macroevolutionary inferences (especially for key taxa), and ancestor-descendant probabilities as our initial analysis (Supplemental Table 1). Thus, the results of each sensitivity analysis are consistent with the macroevolutionary interpretations presented in the main text, indicating our results are robust to a wide range of variation in choice of assumptions and prior distributions.

Method to infer empirical estimates of fossil sampling and specify prior distributions for FBD analysis

To calibrate fossil sampling priors, we used taxonomic occurrence data from the Paleobiology Database to obtain a distribution of per-interval sampling probabilities estimated independently from phylogenetic modeling. We then converted these sampling probabilities to rates, and then used the mean and variance of the Phanerozoic sampling rate distribution to specify a Beta prior on the FBD sampling rate.

All taxonomic occurrence data corresponding to species belonging to Ophiuroidea were downloaded from the Paleobiology Database on August 5, 2021. In total, these data represent 783 occurrences spanning 352 collections of fossil brittle stars. The ophiuroid fossil record

spans nearly the entire Phanerozoic, from the Ordovician Period to the present (~450 million years), and all occurrences were placed in the PBDB's standard 10-million-year time bin intervals.

Using the PBDB dataset of brittle star occurrences, we calculated per-interval sampling probabilities using the part-timer sampling probability based on Alroy's (2008, 2010) three-timer methods, which minimize issues related to edge, Signor-Lipps, or "pull of the Recent" effects (Alroy, 2010). The equation for the part-timer sampling probability (P_s) is:

$$P_s = \frac{N_{3T}}{N_{3T} + N_{PT}}, \qquad (S1)$$

where N_{3T} is the number of *three timers*, defined as taxa sampled in 3 successive time bins (i.e, i, $i + 1$, and $i - 1$), and N_{PT} is the number *part-timers*, defined as taxa sampled immediately before and after a time bin, but not within it (i.e., sampled in bins $i - 1$ and $i + 1$ but not in i) (Alroy 2008, 2010, equation 12). Thus, the part-timer sampling probability is the conditional probability of fossil sampling in an interval given it was extant through the interval.

These empirically estimated values for per-interval sampling probabilities can be used to inform the fossil sampling rate of the FBD analyses by converting probabilities to rates via sampling theory. The net probability of fossil preservation (ξ) from time t_1 to t_2 is:

$$\xi = 1 - e^{-\int_{t1}^{t2} \psi\,(T)\,dT}, \qquad (S2)$$

where $\psi(T)$ is the fossil sampling rate (Foote, 2000, p. 100). If the sampling rate is constant, then this simplifies to:

$$\xi = 1 - e^{-\psi t}. \qquad (S3)$$

Assuming each 10-million-year PBDB time bin (t) is characterized by its own (constant) sampling rate, we can convert the distribution of part-timer sampling probabilities to rates. Equating P_s with ξ and rearranging yields:

$$\psi = \frac{-\ln(1 - \xi)}{t}. \qquad (S4)$$

Now that we have PBDB-based estimates for fossil sampling rates across multiple time bins spanning the Phanerozoic, we can use this information to empirically inform and/or constrain the phylogeny-based parameters. For example, the mean per-interval sampling probability for the Phanerozoic was estimated to be 0.692. Plugging this value into the equation for ψ above, we obtain the sampling rate used in analysis A-4 (Supplemental Table 1). Alternatively, we can use the mean and variance of the Phanerozoic rate distribution to specify a prior on the sampling rate having the same statistical properties as the empirical data. For analyses A-4 and A-5, we placed a beta prior on the sampling rate having the mean and variance of the PBDB-based rate distribution. The shape of a beta distribution is determined by two parameters: α and β. Standard textbooks on probability theory define the mean (μ) and variance (σ^2) of a beta distribution as:

$$\mu = \frac{\alpha}{\alpha + \beta}\,, \qquad (S5)$$

$$\sigma^2 = \frac{\alpha\beta}{(\alpha + \beta)^2 + (\alpha + \beta + 1)}\,. \qquad (S6)$$

With some algebra, these equations can be rearranged to solve for values of α and β in terms of μ and σ^2:

$$\alpha = \left(\frac{1-\mu}{\sigma^2} - \frac{1}{\mu}\right)\mu^2\,, \qquad (S7)$$

$$\beta = \alpha\left(\frac{1}{\mu} - 1\right)\,. \qquad (S8)$$

Thus, one can use these equations to specify the parameters of a beta distribution having a particular mean and variance. The values for α and β in sensitivity analyses A-4 and A-5 were determined by plugging in the values of the mean and variance of the empirical fossil sampling rate distribution determined above from PBDB data.

Supplementary Figure 1: Position of the sampled localities on the island of Gotland, Sweden, modified after Eriksson and Calner (2005).

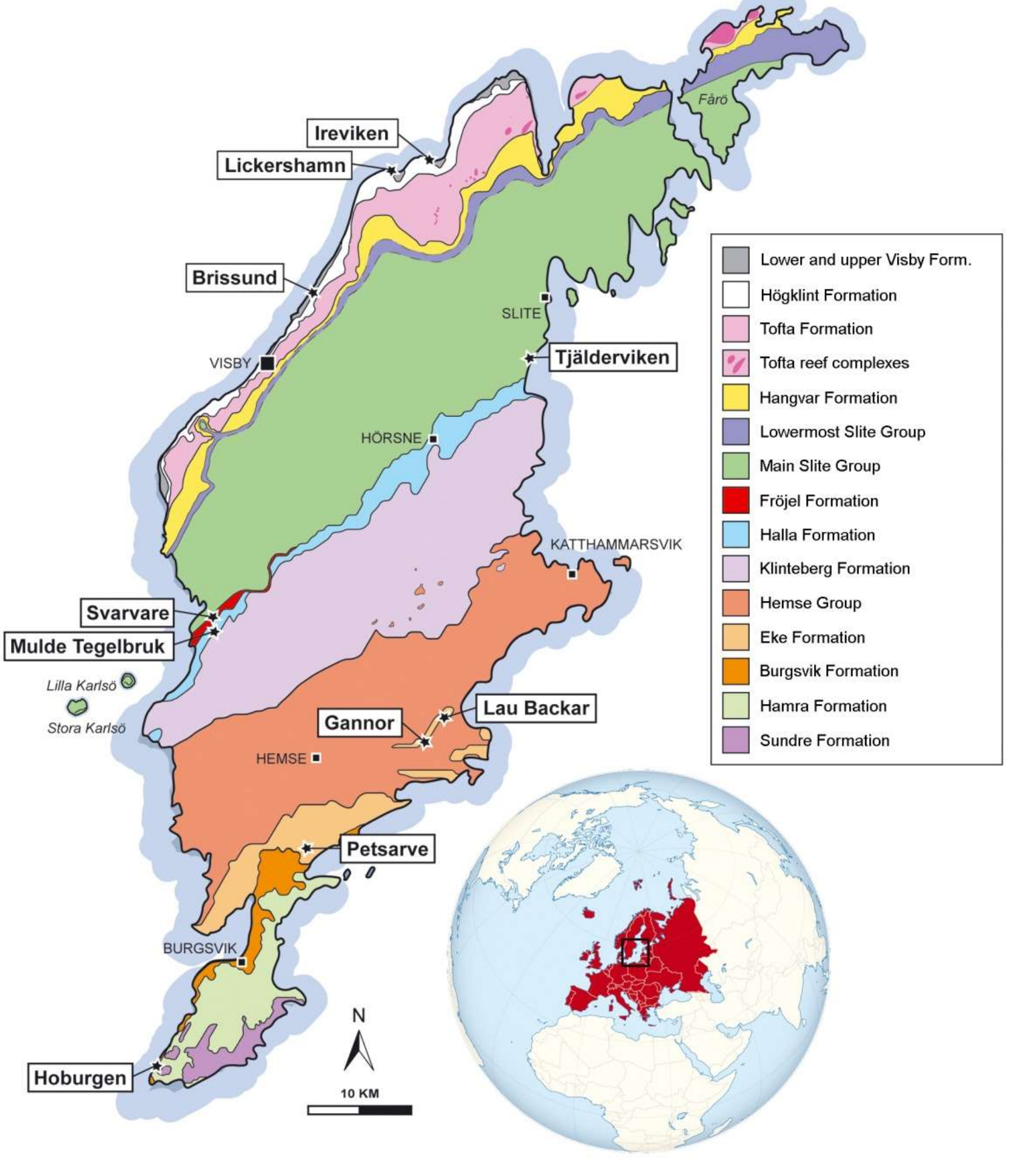

Supplementary Figure 2: Stratigraphic framework of Gotland, with the stratigraphic position of localities sampled for ophiuroids alongside distinguished Silurian events[12].

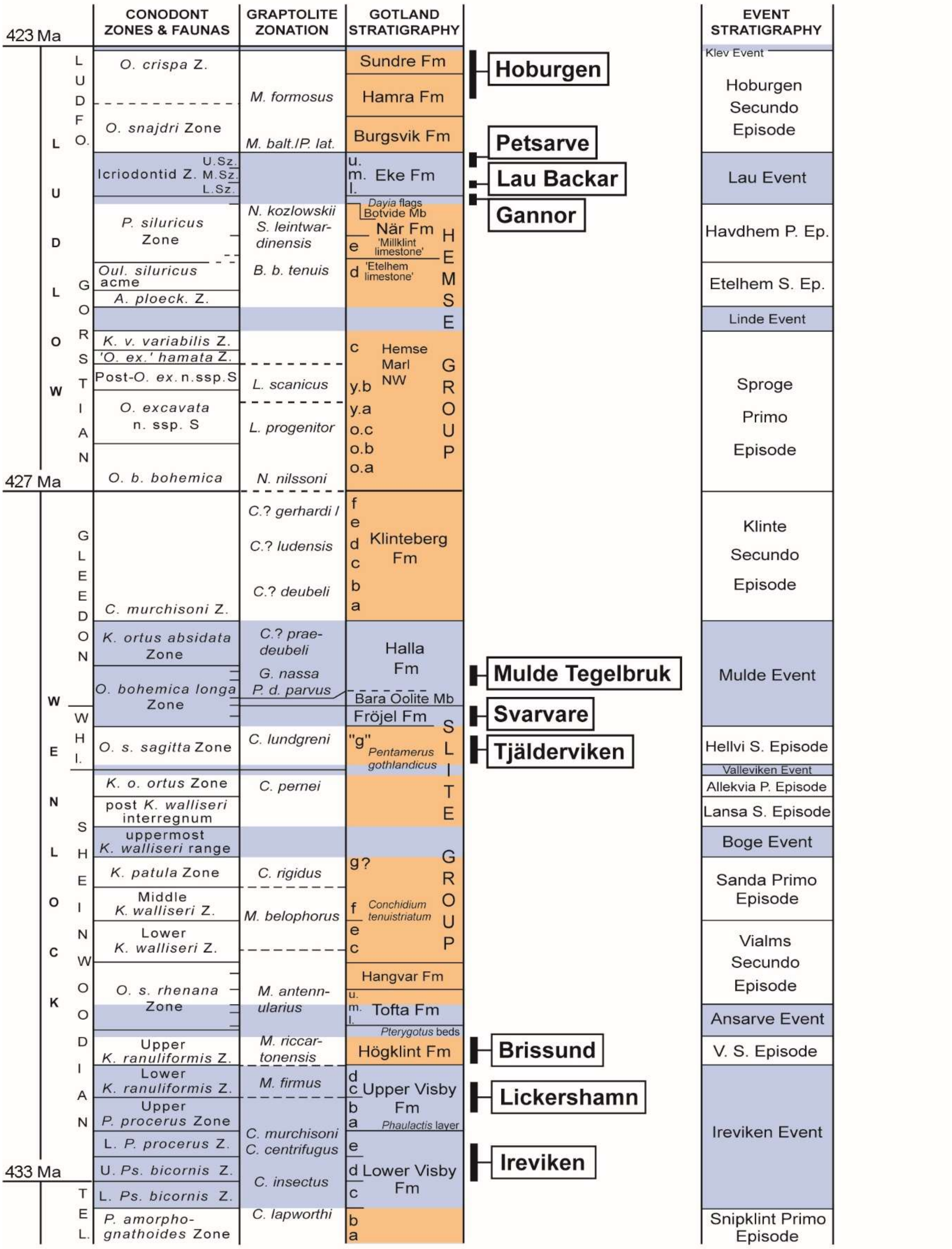

Supplementary Figure 3: *Ophiopetagno paicei* sp. nov. from the Silurian of Gotland. **a-b**, specimen MnhnL OPH101 (all numerical codes in the figure legend refer to specimen numbers in the MnhnL collection). Lateral arm plate in external (**a**) and internal (**b**) views. **c-d**, specimen OPH102. Lateral arm plate in external (**c**) and internal (**d**) views. **e-g**, specimen OPH103. Vertebra in lateral (**e**), dorsal (**f**) and oblique distal-ventral (**g**) views. All scale bars equal 0.1 mm.

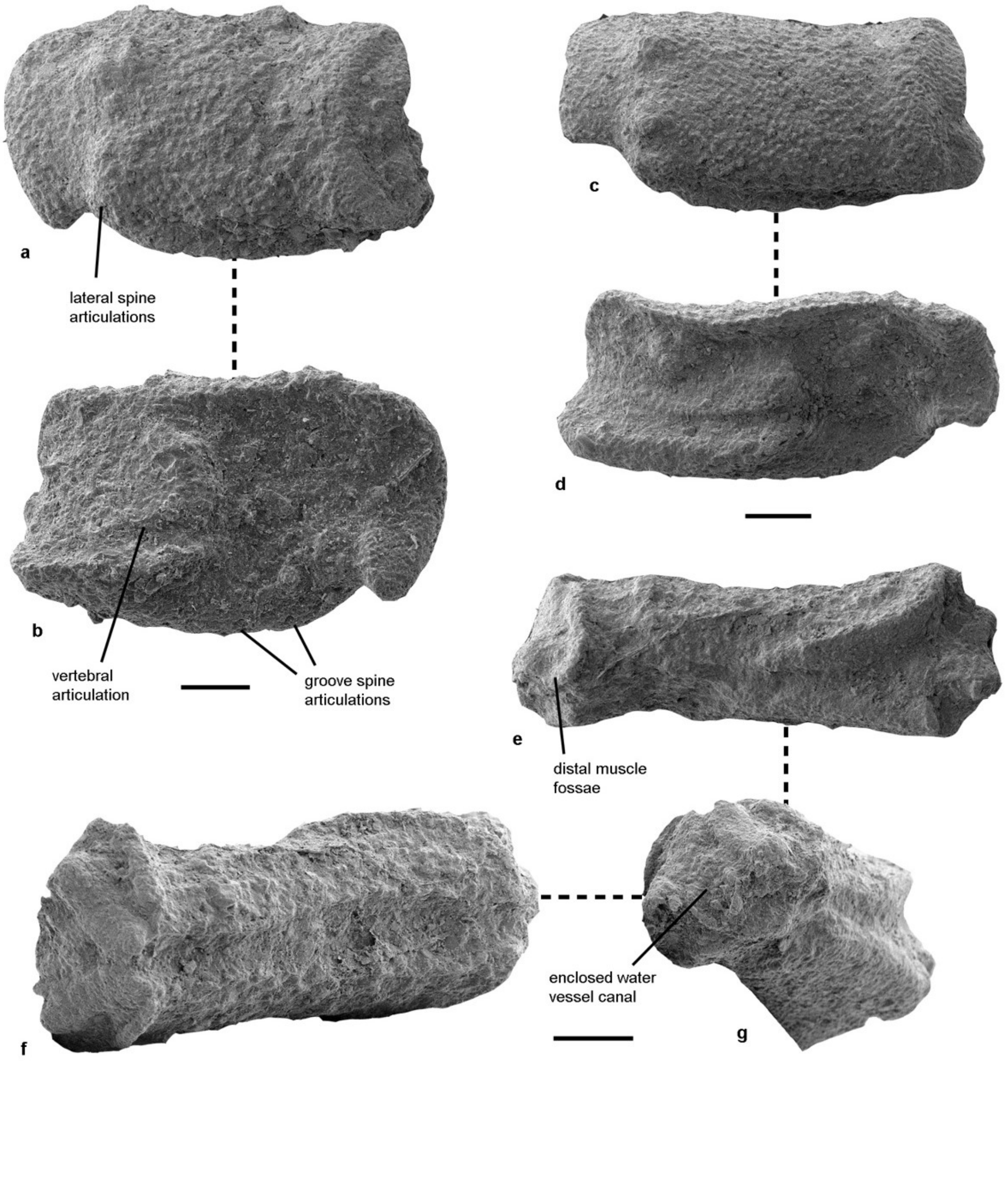

Supplementary Figure 4: *Muldaster haakei* from the Silurian of Gotland, Sweden. **a-c**, specimen MnhnL OPH091 (all numerical codes in the figure legend refer to specimen numbers in the MnhnL collection). Lateral arm plate in external (**a**), ventral (**b**) and internal (**c**) views. **d-e**, specimen OPH094. Lateral arm plate in external (**d**) and internal (**e**) views. **f-i**, specimen OPH095. Vertebra in lateral (**f**), latero-ventral (**g**), dorsal (**h**) and proximal (**i**) views. **j-k**, specimen OPH096. Two articulated arm segments in dorsal (**j**) and ventral (**k**) views. **l**, specimen OPH097. Putative predation regurgitate with various arm plates. All scale bars equal 0.1 mm.

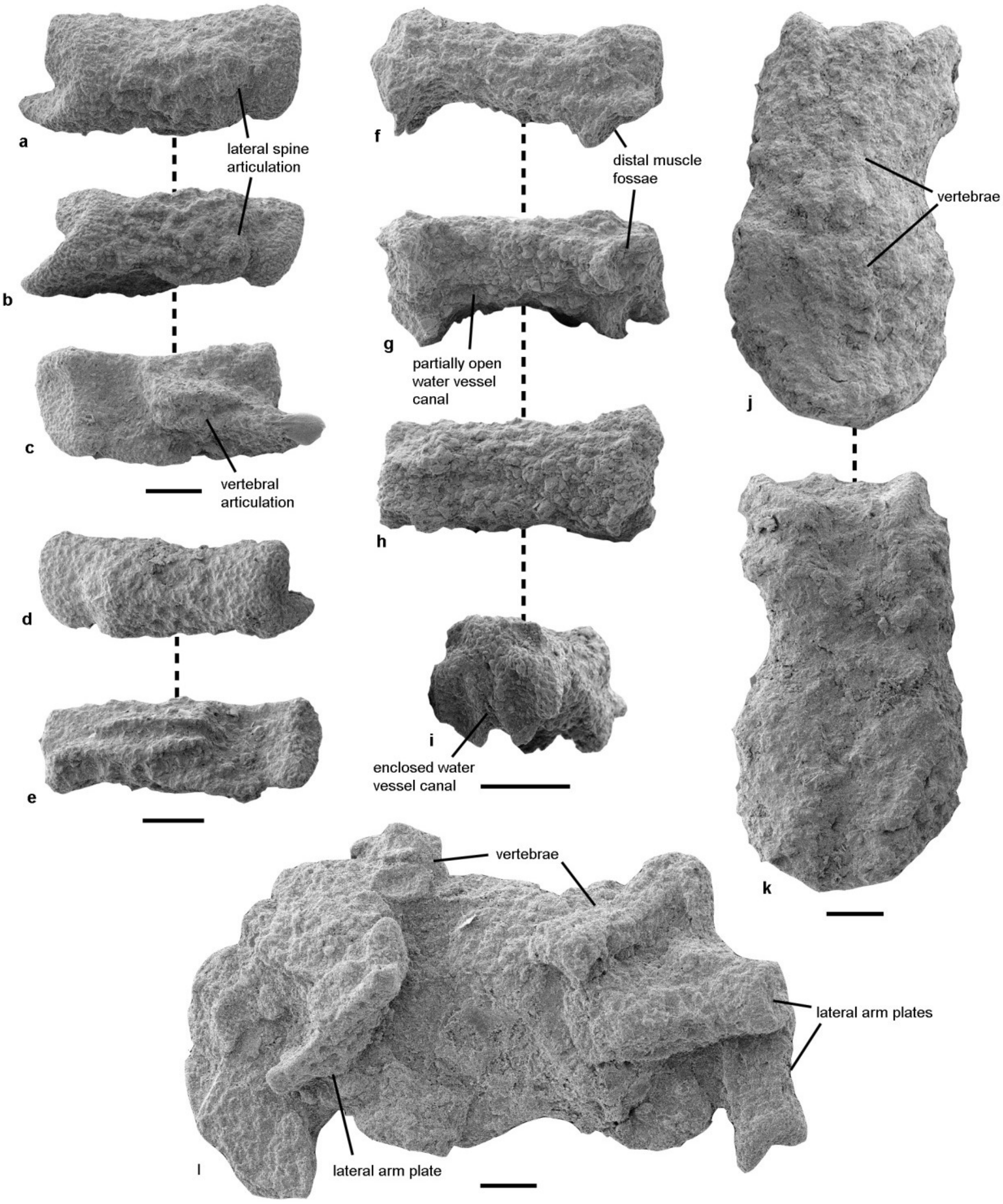

Supplementary Figure 5: Phylogenetic tree of the morphological dataset inferred using the initial unconstrained analysis in MyBayes. Numbers at nodes indicate posterior probabilities.

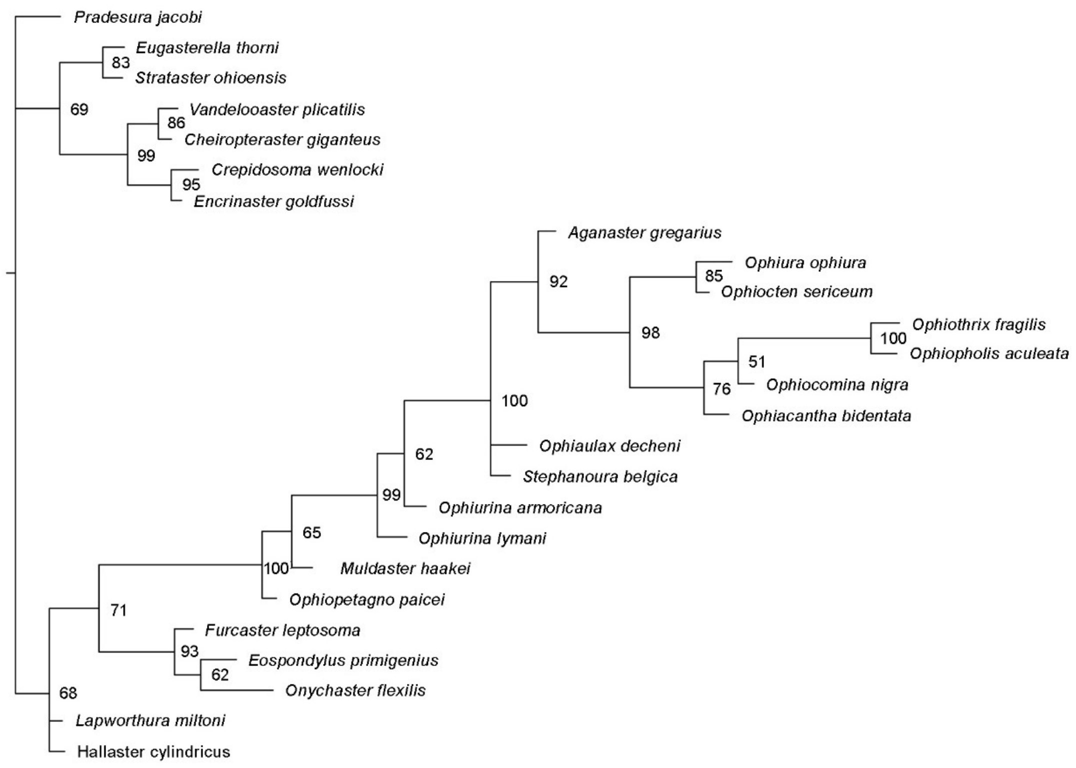

Supplementary Figure 6: Completeness of the ophiuroid fossil record through time. Error bars show +/- 1 standard deviation from the mean.

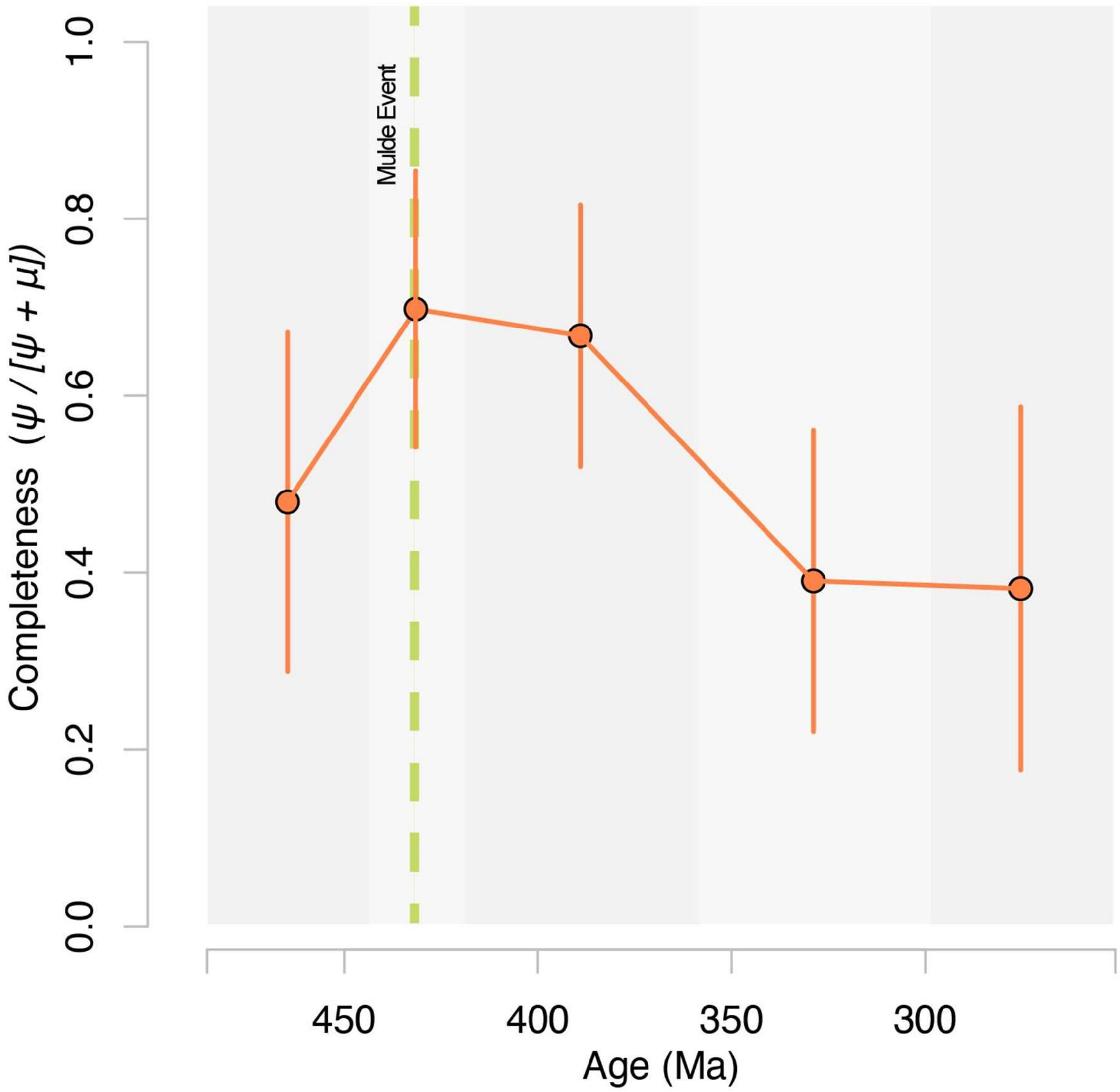

Supplementary Figure 7: Results from the Monte Carlo simulations evaluating the statistical significance of body size decrease across environmental crisis events. Null distributions are shown in gray, and the observed value (i.e., the test statistic Δ_i) is indicated by a red, dotted vertical line. In all cases, Δ_i either falls entirely outside the null distribution or otherwise the p-value well under a significance threshold of an alpha level = 0.05 (Ireviken, p = 0.0003, Mulde, p = 0, Lau, p = 0).

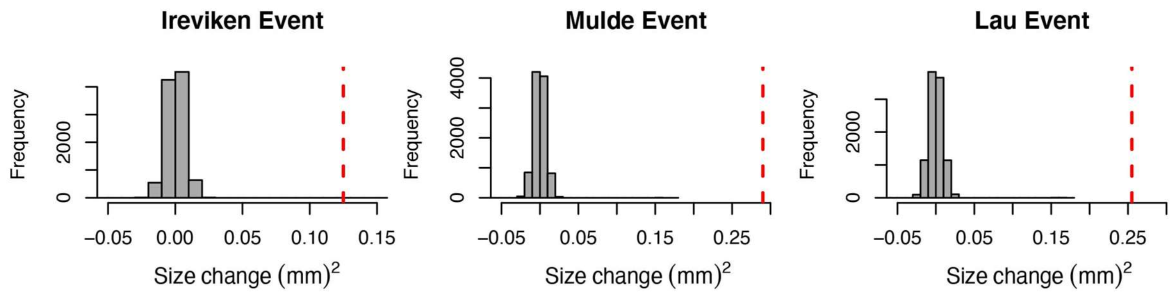

Supplementary Table 1: Average and standard deviation for lateral arm plate surface area measures.

Localites	Number of plates	Average surface area (mm^2)	Standard deviation (mm^2)
Hoburgen	1	0.903	N/A
Petsarve	228	0.257	0.037
Lau Backar	25	0.386	0.082
Gannor	22	0.249	0.072
Mulde	297	0.5	0.015
Svarvare	96	0.117	0.037
Tjaelderviken	16	0.34	0.06
Brissund	350	0.099	0.049
Lickershamn	39	0.294	0.089
Ireviken	11	0.224	0.067

Supplementary Table 2: Variation in prior distributions and results of sensitivity analyses. "Constrained" vs. "Unconstrained" reflects whether or not the topology was set to be consistent with the undated analysis or left free to vary across the analysis. "Variable" vs. "Constant" indicates whether a single-rate FBD process vs. a time-varying model was used, where separate rates are estimated for each geological stage. Numbers in columns corresponding to taxon names represent the frequency (interpreted here as a posterior probability) in which that taxon is placed as a sampled ancestor across the posterior distribution of trees. "Main analysis" refers to the tip-dated analysis presented in the main text, and is presented here for comparison.

Analysis	Topology	FBD rates	Sampling rate prior	IGR variance prior	Base rate of the clock prior	*O. paicei*	*M. haakei*
Main analysis	Constrained	Variable	Beta(2, 2)	Uniform(0.001, 200)	Normal(0.0025, 0.1)	1	1
A-1	Unconstrained	Variable	Beta(2, 2)	Uniform(0.001, 200)	Normal(0.0025, 0.1)	1	1
A-2	Constrained	Constant	Beta(2, 2)	Uniform(0.001, 200)	Normal(0.0025, 0.1)	1	1
A-3	Constrained	Variable	Fixed to 0.1177655	Uniform(0.001, 200)	Normal(0.0025, 0.1)	0.998	1
A-4	Constrained	Variable	Beta(0.0704723, 0.5279398)	Uniform(0.001, 200)	Normal(0.0025, 0.1)	1	1
A-5	Unconstrained	Variable	Beta(0.0704723, 0.5279398)	Uniform(0.001, 200)	Normal(0.0025, 0.1)	0.998	0.999
A-6	Unconstrained	Variable	Beta(2, 2)	Exp(274.8)	Lognormal(-5.348, 1.115)	0.998	0.997

Supplementary References

Alroy, J., 2008. Dynamics of origination and extinction in the marine fossil record. *Proceedings of the National Academy of Sciences*, *105*, pp.11536-11542.

Alroy, J., 2010. Fair sampling of taxonomic richness and unbiased estimation of origination and extinction rates. *The Paleontological Society Papers*, *16*, pp.55-80.

Eriksson, M.E. and Calner, M., 2005. The Dynamic Silurian Earth - Subcommission on Silurian Stratigraphy Field Meeting 2005. *SGU, Rapporter och Meddelanden* 121.

Foote, M. and Raup, D.M., 1996. Fossil preservation and the stratigraphic ranges of taxa. *Paleobiology*, *22*(2), pp.121-140.

Foote, M., 2000. Origination and extinction components of taxonomic diversity: general problems. *Paleobiology*, *26*(S4), pp.74-102.

Lewis, P.O., 2001. A likelihood approach to estimating phylogeny from discrete morphological character data. *Systematic biology*, *50*(6), pp.913-925.

Louca, S., McLaughlin, A., MacPherson, A., Joy, J.B. and Pennell, M.W., 2021. Fundamental identifiability limits in molecular epidemiology. *Molecular Biology and Evolution*.

Matzke, N.J. and Wright, A., 2016. Inferring node dates from tip dates in fossil Canidae: the importance of tree priors. *Biology Letters*, *12*(8), p.20160328.

Ronquist, F., Klopfstein, S., Vilhelmsen, L., Schulmeister, S., Murray, D.L. and Rasnitsyn, A.P., 2012. A total-evidence approach to dating with fossils, applied to the early radiation of the Hymenoptera. *Systematic Biology*, *61*(6), pp.973-999.

Wagner, P.J., 2012. Modelling rate distributions using character compatibility: implications for morphological evolution among fossil invertebrates. *Biology Letters*, *8*(1), pp.143-146.
