# Supplementary material for: Miniaturization during a Silurian environmental crisis generated the modern brittle star body plan
Source: Commun Biol. 2022 Jan 10;5:14. doi: 10.1038/s42003-021-02971-9 (PMC8748437; doi:10.1038/s42003-021-02971-9)
Supplement: Supplementary file 3 — Description of Additional Supplementary Files [file 42003_2021_2971_MOESM3_ESM.pdf]

Description of Additional Supplementary Files

File name: Supplementary Data 1.
Description: Character list and matrix used for the Bayesian-inference analysis.

File name: Supplementary Data 2.
Description: Lateral arm plate surface area used for the body size graph.
